# Roles of Hydrogen Sulfide (H2S) as a Potential Therapeutic Agent in Cardiovascular Diseases: A Narrative Review

**DOI:** 10.7759/cureus.64913

**Published:** 2024-07-19

**Authors:** Kazi N Islam, Ivan D Nguyen, Rahib Islam, Humza Pirzadah, Hassan Malik

**Affiliations:** 1 Department of Agricultural Research Development Program, Central State University, Wilberforce, USA; 2 School of Medicine, Louisiana State University Health Sciences Center, New Orleans, USA

**Keywords:** atherosclerosis, arrhythmia, heart failure (hf), cardiac remodeling, ischemic-reperfusion injury, myocardial infarction (mi), cardiovascular disease, heart disease, hydrogen sulfide (h2s)

## Abstract

Cardiovascular disease (CVD) stands as one of the leading causes of morbidity and mortality worldwide, and the continued search for novel therapeutics is vital for addressing this global health challenge. Over the past decade, hydrogen sulfide (H₂S) has garnered significant attention in the field of medical research, as it has been proven to be a cardioprotective gaseous signaling molecule. It joins nitric oxide and carbon monoxide as endogenously produced gasotransmitters. As for its mechanism, H₂S functions through the posttranslational addition of a sulfur group to cysteine residues on target proteins in a process called sulfhydration. As a result, the observed physiological effects of H₂S can include vasodilation, anti-apoptosis, anti-inflammation, antioxidant effects, and regulation of ion channels. Various studies have observed the cardioprotective benefits of H₂S in diseases such as myocardial infarction, ischemia-reperfusion injury, cardiac remodeling, heart failure, arrhythmia, and atherosclerosis. In this review, we discuss the mechanisms and therapeutic potential of H₂S in various CVDs.

## Introduction and background

The cardiovascular system, composed of the heart and a network of blood vessels, works together to distribute oxygen and nutrients throughout the body. When anomalies and abnormalities occur within this system, they are collectively referred to as cardiovascular disease (CVD) or heart disease (HD) [1]. CVD is one of the leading causes of premature death worldwide, and as a result, it is one of the main factors that have a role in the high socio-economic burden from things such as hospital bills from these conditions in the general population [2]. Furthermore, to be able to effectively treat HD as a practicing clinician, it is important to be aware of the epidemiology and overall statistics of the disease. As time passes, not only does the incidence rise but the burden of the disease as well. In 1990, there were a total of around 271 million cases of CVD, as reported by the Global Burden of Disease, which nearly doubled to around 523 million in recent years [3]. Even in places such as Europe, CVDs are responsible for around half of all deaths [4]. Even disregarding this unprecedented rise in people affected by this disease, this number is further expected to increase and is projected to increase by the year 2060, with various cardiovascular risk factors being measured, such as diabetes mellitus. The number of people with the disease is projected to increase by 39%, from 39.2 million to 54.6 million, hypertension by 27.2% (127.8-162.5 million); dyslipidemia by 27.5% (98.6-125.7 million), and obesity by 18.3% (106.3-125.7 million). In addition, projected prevalence will similarly increase compared with 2025 for ischemic HD by 31.1% (21.9-28.7 million), heart failure (HF) by 33.0% (9.7-12.9 million), myocardial infarction (MI) by 30.1% (12.3-16.0 million), and stroke by 34.3% (10.8-14.5 million) [5].

## Review

Mechanisms of hydrogen sulfide in cardiovascular diseases

Nitric oxide (NO) and carbon monoxide (CO) are both endogenously produced gasotransmitters and have been extensively studied for their cardioprotective effects mediated through vasodilation [6,7]. More recently, hydrogen sulfide (H_2_S) has emerged as another endogenously produced gaseous signaling molecule with potent cardiovascular protective benefits [8,9]. Its therapeutic benefits have been noted in a spectrum of diseases such as MI, ischemia-reperfusion injury, cardiac remodeling, HF, arrhythmia, and atherosclerosis [10]. Both NO and H_2_S are known to increase heme oxygenase 1 (HO-1) levels, an enzyme that produces CO [11,12].

The endogenous production of H_2_S is attributed to three main enzymes: cystathionine b-synthase (CBS), 3-mercapto pyruvate sulfur transferase (3-MST), and cystathionine gamma-lyase (CSE) [13]. In the brain, 3-MST is the major source of production for the neuromodulator H_2_S. The antioxidant properties of H_2_S allow the molecule to play a role as a neuroprotective agent against oxidative stress [14,15]. Additionally, 3-MST is present in both mitochondria and vasculature, while CBS is primarily localized to the brain and central nervous system. Notably, the predominant production of H_2_S in vascular smooth muscle is attributed to CSE [13]. As for substrates, sulfur-containing amino acids such as L-homocysteine and L-cysteine are essential for enzymatic production [9]. It is important to note that non-enzymatic production of H_2_S in the bloodstream is possible through cysteine catalysis with iron and vitamin B6 [16].

The mechanisms underlying H_2_S signaling occur through sulfhydration or persulfidation, both of which entail the post-translational modification of cysteine residues on target proteins [17]. Sulfhydration has been associated with a multitude of physiological processes, such as the regulation of mitochondrial metabolism, stress signaling, antioxidant properties, vasorelaxation, and anti-inflammatory properties [18,19]. In exploring the role of H_2_S in CVDs, we hope to further elucidate its potential therapeutic applications and contribute to novel treatment strategies that aim to leverage its cardioprotective mechanisms. Recently, it has been demonstrated that one mechanism by which H_2_S exerts cytoprotective action is via the upregulation of cellular antioxidants in a nuclear-factor-E2-related factor-2 (Nrf2)-dependent manner [11]. Nrf2 regulates the gene expression of a number of enzymes, such as GPx1 and HO-1, that serve to detoxify pro-oxidative stressors [20] by binding to the antioxidant response element found in the gene promoter region [11].

Causes and risk factors of cardiovascular diseases

There are various causes and risk factors when it comes to CVD. In the context of causes and risk factors for CVD, significant behavioral factors include an unhealthy diet, a lack of physical activity, tobacco use, and excessive alcohol consumption. These behaviors often manifest in individuals as elevated blood pressure, glycemia, and lipid levels, as well as overweight or obesity. Such "intermediate risk factors" can be assessed in primary care settings and signify an increased susceptibility to heart attacks, strokes, HF, and related complications [21].

Strategies like quitting smoking, reducing dietary salt intake, consuming more fruits and vegetables, engaging in regular physical activity, and moderating alcohol consumption have proven effective in mitigating CVD risk [2]. Policies promoting environments conducive to healthy choices, which are both affordable and accessible, are crucial for encouraging and sustaining healthy behaviors among individuals [22]. Such policies could include initiatives like grocery store tours led by local medical students to promote healthy food choices, group workout sessions to encourage physical activity, and educational classes to raise awareness about the risks of CVD [23].

The underlying determinants of CVDs are deeply rooted in broader social, economic, and cultural shifts such as globalization, urbanization, and population aging. Additional factors contributing to CVD risk include poverty, stress, and genetic predispositions [24].

Furthermore, pharmacological interventions targeting conditions like hypertension, diabetes, and high lipid levels are essential for reducing cardiovascular risk and preventing heart attacks and strokes in affected individuals [25]. These interventions complement lifestyle modifications and play a crucial role in managing CVD risk factors effectively.

Another major risk factor for CVD is atherosclerosis. Atherosclerosis is defined as the accumulation of cholesterol plaque within artery walls, leading to the hardening of such walls. Endothelial dysfunction is labeled as one of the most important factors contributing to the pathogenesis of CVD [26].

Atherosclerosis begins with endothelial dysfunction and the retention of low-density lipoprotein (LDL) in the intima [27]. Modified LDLs, along with other atherogenic factors, activate endothelial cells and recruit monocytes into the intima [26]. Monocytes and vascular smooth muscle cells capture modified LDLs, leading to foam cell formation and fatty streak formation, characterized by lipid accumulation [28]. Endothelial dysfunction disrupts vascular homeostasis, predisposing vessel walls to vasoconstriction, lipid infiltration, leukocyte adhesion, platelet activation, and oxidative stress, initiating an inflammatory response and fatty streak formation. Disturbed hemodynamic forces cause turbulent flow, promote endothelial dysfunction, and facilitate lipoprotein infiltration into the intima [29]. Shear stress affects endothelial gene expression, upregulating atherogenic genes like MCP-1 and PDGFs while promoting the expression of genes that induce cell-cycle growth arrest or increase antioxidant capacity [30]. Undisturbed laminar flow upregulates endothelial NO synthase (eNOS), enhancing NO synthesis. This complex interplay contributes to the progression of atherosclerosis.

Risk factors for atherosclerosis include hypertension, cigarette smoking, and diabetes mellitus [31]. H_2_S has been shown to have several therapeutic effects when it comes to atherosclerosis, such as maintaining endothelial cell dysfunction [32]. In a study conducted by Tian et al., it was seen that the depletion of H_2_S resulting from CSE depletion contributes significantly to the onset of endothelial dysfunction. Their research indicates that in the context of CSE/H_2_S deficiency-associated endothelial dysfunction, the MAPK/TXNIP (thioredoxin interacting protein) signaling pathway plays a pivotal role. Moreover, the CSE/H_2_S pathway appears to exert protective effects against the development of uremia-accelerated atherosclerosis by modulating the expression of eNOS through the conventional protein kinase C βII/Akt signaling pathway. In addition to CSE, CBS also influences atherosclerosis progression. Mutations in the CBS gene contribute to endothelial dysfunction, thereby contributing to the development of cardiovascular and neurovascular diseases. The CBS/H_2_S pathway interacts with mitochondrial function and endoplasmic reticulum-mitochondrial tethering, thereby affecting endothelial cell dysfunction-related pathologies. However, the role of 3-MST in maintaining endothelial cell function in atherosclerosis remains to be thoroughly investigated. Taken together, the levels of H_2_S and CSE/CBS/3-MST could serve as potential biomarkers and therapeutic targets for patients with atherosclerosis [33].

Myocardial infarction

In the realm of cardiovascular health, MI, also known as a "heart attack," stems from a decrease or complete halt in blood flow to a segment of the myocardium. While some instances of MI can occur without noticeable symptoms, others can provoke severe hemodynamic decline and result in sudden fatalities. Predominantly rooted in underlying coronary artery disease, which stands as the primary cause of mortality in the United States, MI unfolds with the obstruction of coronary arteries, depriving the myocardium of essential oxygen. Prolonged oxygen deprivation precipitates the demise and necrosis of myocardial cells. Symptoms may manifest as chest discomfort or pressure, extending to the neck, jaw, shoulder, or arm. In conjunction with patient history and physical examination, the presence of myocardial ischemia might coincide with ECG alterations and heightened biochemical markers such as cardiac troponins [34]. Furthermore, most deaths that occur due to MI are acute. In examining risk factors for CVD, it's important to consider both nonmodifiable and modifiable factors. Nonmodifiable risk factors encompass characteristics beyond individual control, including gender, age, family medical history, and even male pattern baldness. Conversely, modifiable risk factors offer avenues for intervention and lifestyle adjustments. These include smoking, dyslipidemia, diabetes mellitus, hypertension, obesity, a sedentary lifestyle, poor oral hygiene, the presence of peripheral vascular disease, and elevated levels of homocysteine.

In addition to these factors, there are other causes of MI that merit consideration. Trauma, vasculitis, substance abuse (such as cocaine), coronary artery anomalies, coronary artery emboli, aortic dissection, and conditions that place excessive demand on the heart (such as hyperthyroidism and anemia) can all contribute to the onset of MI and related cardiovascular complications. Understanding and addressing these diverse risk factors is crucial for effective prevention and management strategies in CVD care [35].

H_2_S has also been shown to have several protective factors in MI, such as in the form of garlic [36]. Its cardiovascular benefits have been extolled for centuries, though the specific cytoprotective mechanisms remained elusive until recently. It is now understood that garlic's cardiovascular advantages stem partly from allicin, also known as diallyl thiosulfinate. Allicin metabolizes into three primary H_2_S-producing compounds: diallyl sulfide, diallyl disulfide, and diallyl trisulfide (DATS). These metabolites have undergone extensive investigation, revealing their cardioprotective properties mediated by H_2_S elaboration. Studies by Predmore et al. highlighted the efficacy of DATS in inducing cardioprotection in an in vivo model of MI/reperfusion injury. Administering 200 μg/kg DATS via intravenous or intraperitoneal routes before reperfusion during ischemia effectively reduces myocardial infarct size and circulating troponin-I levels while enhancing cardiac function through H_2_S level restoration post-ischemia. Furthermore, DATS demonstrates concentration-dependent reductions in mitochondrial respiration and notably improves mitochondrial coupling post-reperfusion. Additionally, DATS promotes eNOS activation, consequently enhancing NO bioavailability, thus contributing to its cardioprotective effects [37]. It has also been reported that NO ameliorates myocardial dysfunction via H_2_S production in a mouse model [38].

Ischemia-reperfusion injury

Following MI, reperfusion of blood is essential for replenishing the ischemic tissue, but paradoxically, it can also cause further damage to the same tissue it replenishes. The mechanism of ischemia-reperfusion injury is caused by increased oxidative stress from reactive oxygen species (ROS), increased calcium leading to myocyte hypercontraction, rapid restoration of pH, and inflammatory damage upon reperfusion of ischemic tissue [39]. Modern medicine has solved the problem of cardiac ischemia with the gold standard method of percutaneous coronary intervention, but there has been no firm answer to prevent post-infarction damage caused by ischemia-reperfusion injury [40,41].

H_2_S has been observed to protect against myocardial ischemia-reperfusion injury through its role as an antioxidant, anti-apoptotic, anti-inflammatory, and ability to protect the mitochondria [42]. One mechanism for H_2_S protecting against ROS is through downregulation of NF-kB and the JAK2/STAT3 pathway. A reduction in these pathways resulted in decreased production of ROS [43]. Additionally, the downregulation of NF-kB serves as a mechanism of H_2_S in reducing ischemia-reperfusion-induced inflammatory pathways [44]. Another method for H_2_S to combat ROS in ischemia-reperfusion injury is by upregulating antioxidants, HO-1 and Trx1, through the activation of the transcription factor Nrf2 [11]. Nrf2 regulates the production of enzymes responsible for maintaining and decreasing oxidative stress in the body [20,45]. Likewise, Nrf2 activation by H_2_S favors anti-apoptotic processes by increasing the expression of protective molecules such as heat shock proteins and Bcl-2 [11].

An in vivo mouse study by Elrod et al. (2007) reported that H_2_S treatment reduced reperfusion infarction size, preserved left ventricular function, and protected mitochondrial integrity after induction of myocardial ischemia-reperfusion injury [46]. In a similar study on isolated rat hearts by Ravindran et al. (2017), sodium thiosulfate, an H_2_S precursor, also demonstrated a decrease in ischemia-reperfusion infarction size and recovery of the failing heart. The study observed a marked decrease in the apoptotic enzyme, caspase-3 [47]. A more recent study by Sun et al. (2018) discovered that long-term and slow-releasing H_2_S improved cardiac allograft preservation and improved survival and function after eight weeks of transplantation. Although this study was not directly related to pathological ischemia-reperfusion injury, it still highlights the importance of H_2_S in cardioprotection prior to a reperfusion event [48]. Finally, a study by Jeddi et al. (2020) revealed that an intermediate dose of NaSH decreased infarction size, decreased oxidative stress, and improved hemodynamics in rats following an induced myocardial ischemia-perfusion injury [49].

Cardiac remodeling

Cardiac remodeling is a pathological process that involves either an injury to the heart or volume overload, causing a permanent alteration in cardiac structure, shape, and function. It can be divided into the stages of initial ischemia, necrosis and apoptosis, inflammation, and permanent changes to the extracellular matrix. Furthermore, these detrimental changes to the heart can present as cardiomyocyte hypertrophy, loss of cardiomyocytes, or invasion of cardiac tissue by myocardial fibrosis [50]. Myocardial fibrosis is defined by the pathological deposition of extracellular matrix proteins by myofibroblasts. Although fibrosis is a naturally occurring process for healing and repairing damaged tissues, fibrosis in the heart replaces cardiomyocytes with nonfunctional scar tissue. As a result, the fibrosis prevents cardiac rupture, but diastolic or systolic function will be impaired [51].

On a molecular level, it has been discovered that chronic activation of angiotensin II has been linked to the induction of cardiac remodeling. Thus, the current drugs employed to treat cardiac remodeling act on the renin-angiotensin-aldosterone system, and the preferred pharmacologic agents include angiotensin-converting enzyme inhibitors and angiotensin receptor blockers [52].

In a similar way, H_2_S may alleviate cardiac fibrosis and hypertrophy through inhibition of angiotensin II activity in cardiac tissue [53,54]. Angiotensin II induces cardiac remodeling through the activation of the KLF5 gene [53]. KLF5 is responsible for orchestrating cardiovascular remodeling through cardiac hypertrophy, fibrosis, arterial wall thickening, and angiogenesis. Heterozygous KLF5-knockout mice showed decreased cardiac remodeling after induced vascular injury [53], and in a similar way, H_2_S has been found to decrease myocardial hypertrophy through mechanisms of downregulating KLF5 gene expression [44]. In a study by Huang et al. (2012), researchers examined the effects of H_2_S on cardiac volume overload by inducing abdominal aortic coarctation in rats, and the results showed that rats treated with H_2_S had smaller cardiomyocytes and less fibrosis [54]. Furthermore, in a study by Su et al. (2021), atrial fibrosis induced by atrial fibrillation was reduced in mice that were exposed to H_2_S [55].

Although there is already an abundance of drugs on the market designed to target the renin-angiotensin-aldosterone system, it would be interesting to compare the efficacy of H_2_S against the current therapeutics in treating cardiac remodeling. It can also be of interest to observe the effects of H_2_S in conjunction with current drugs to see if the benefits can be maximized.

Heart failure

In accordance with the definitions provided by the American College of Cardiology (ACC) and the American Heart Association (AHA), congestive HF is characterized as a multifaceted clinical syndrome arising from structural or functional abnormalities that hinder the effective filling or ejection of blood from the ventricles [56].

The etiology of HF encompasses a broad spectrum of factors. Management strategies generally focus on alleviating systemic and pulmonary congestion and stabilizing hemodynamic status, irrespective of the underlying cause. Effective HF treatment necessitates a comprehensive approach involving patient education, optimal medication use, and the reduction of acute exacerbations [57].

The ACC and AHA categorize HF into stages: stage A (HF risk factors present without symptoms or structural HD); stage B (structural HD present without HF symptoms); stage C (structural HD with current or past HF symptoms); and stage D (refractory symptoms despite guideline-directed medical therapy) [58].

H_2_S has been shown to have several therapeutic effects when it comes to treating several CVDs, such as HF. H_2_S donors have been demonstrated to mitigate the intensity of HF induced by pressure overload through the modulation of vascular tone, facilitation of angiogenesis, inflammation reduction, mitigation of oxidative stress, and apoptosis downregulation [59]. There have been several studies done showcasing the various therapeutic effects of H_2_S. For example, in HF, decreased circulating H_2_S levels observed in Cth−/− mice were directly correlated with cardiac dilatation and dysfunction. Conversely, the administration of exogenous H_2_S therapy exhibited cardioprotective effects by upregulating the VEGF-AKT1-eNOS-NO-cGMP pathway, consequently preserving mitochondrial function and enhancing myocardial vascular density. Treatment with the sulfur-donating drug SG1002 in Cth−/− mice similarly augmented myocardial vascular density and ameliorated cardiac remodeling and function through the same pathway. Subsequent research revealed SG-1002's efficacy in elevating circulating H_2_S and NO bioavailability while mitigating B-type natriuretic peptide levels, indicative of reduced cardiomyocyte stress and left ventricular dysfunction, in HF patients with reduced ejection fraction (NYHA class II-III) [19].

Cardiac arrhythmias

It is estimated that 1.5% to 5% of the general population suffers from cardiac arrhythmia. Arrhythmia is defined as any heart rhythm that deviates from the normal sinus rhythm, and these irregular heart rhythms arise from electrical abnormalities and defects along the conduction pathways in the heart [60].

H_2_S can affect arrhythmia through the regulation of ion channels. In two separate rat studies by Sun et al. (2008) and Dai et al. (2019), H_2_S was shown to inhibit L-type calcium channels in both smooth muscle vasculature and cardiomyocytes. The inhibition resulted in decreased intracellular calcium with the benefits of vasorelaxation and increased action potential duration [61,62]. Through inhibition of L-type calcium channels, H_2_S has the potential to serve as an antiarrhythmic agent by reducing automaticity and slowing atrioventricular nodal conduction. Class IV antiarrhythmics function similarly with inhibition of L-type calcium channels in the heart, but these medications are riddled with adverse side effects [63]. Safer drug alternatives are always being sought out; however, in terms of H_2_S, many studies are still needed to analyze if it has any efficacy in directly treating arrhythmia and if the side-effect profile is more favorable than current drugs.

Another antiarrhythmic effect of H_2_S is protection against high-frequency pacing, which is a common feature of atrial fibrillation. A study on mouse cardiomyocytes by Al-Owais et al. (2023) showed that H_2_S prevented cellular and electrophysiological remodeling through inhibition of the ultra-rapid rectifying potassium channel and the Kv1.5 channel [64]. In atrial fibrillation and HF, dysregulation of potassium channels is a common cause of disease progression [65]. Additionally, H_2_S was shown to increase the bioavailability of NO, and this increase in NO and S-nitrosylation may serve as another possible mechanism for treating atrial fibrillation [64].

In a study by Whiteman et al. (2017), H_2_S decreased the likelihood of reperfusion-induced arrhythmias in vivo in rat models. It is important to note that the study used both mitochondria targeting H_2_S and a global H_2_S donor, and the protective benefits were only present in H_2_S that specifically targeted mitochondria [66]. The evidence about H_2_S protecting against arrhythmia in vivo is truly promising, and along with the multitude of studies about its ion-regulating mechanisms, H_2_S has the potential to be a therapeutic agent for arrhythmic disorders in the future.

Limitations

Despite the promising therapeutic potential of H_2_S in CVD, several limitations and challenges must be addressed to translate these findings into clinical practice. Most of the current research on H_2_S has been conducted using animal models or in vitro studies. While these studies provide valuable insights, their applicability to human physiology and pathology remains uncertain. There is a pressing need for well-designed clinical trials to evaluate the safety and efficacy of H_2_S-based therapies in humans.

The therapeutic effects of H_2_S can vary significantly depending on the source and method of H_2_S delivery. Different H_2_S donors have distinct pharmacokinetic and pharmacodynamic properties, which can influence their effectiveness and safety profiles. Standardization of H_2_S delivery methods is essential to ensuring consistent and reproducible results. Although the cardioprotective mechanisms of H_2_S have been extensively studied, our understanding is still incomplete. More research is needed to fully elucidate the molecular pathways through which H_2_S exerts its effects, particularly in the context of different cardiovascular conditions such as MI, HF, and atherosclerosis [67].

The long-term safety of H_2_S therapy is not yet well-established. Chronic administration of H_2_S or its donors may have unforeseen adverse effects, and comprehensive toxicity studies are required to assess the potential risks associated with prolonged use. H_2_S may interact with other medications commonly used in the treatment of CVD, potentially leading to adverse effects or reduced efficacy. Understanding these interactions is crucial for the safe integration of H_2_S therapy into existing treatment regimens. The development and approval of H_2_S-based therapeutics will face regulatory hurdles. Demonstrating the safety, efficacy, and quality of these therapies to regulatory bodies will require substantial evidence from rigorous clinical studies [68].

Furthermore, there is variability in the reported physiological concentrations of H_2_S, which range from nanomolar to micromolar levels. This variability is due to differences in detection methods and the specific tissues analyzed, complicating the establishment of standard therapeutic doses. Finally, while some studies highlight the beneficial anti-inflammatory and anti-oxidative effects of H_2_S, other studies suggest it may also have pro-inflammatory actions under certain conditions. These contradictory findings necessitate further investigation to clarify the contexts in which H_2_S exerts its beneficial versus harmful effects [69].

Addressing these limitations through continued research and collaboration between scientists, clinicians, and regulatory authorities will be essential to harnessing the full therapeutic potential of H_2_S in CVD management.

## Conclusions

The role of H_2_S as a potential therapeutic in various CVDs, including myocardial infarction, ischemia-reperfusion injury, cardiac remodeling, HF, arrhythmia, and atherosclerosis, has been demonstrated in various studies. It is important to note that most of the studies on H_2_S have been done on non-human models. Furthermore, it is necessary to continue elucidating all the physiological effects and interactions of H_2_S. As H_2_S begins moving towards the clinical stages, thorough studies observing the safety and adverse effect profiles of H_2_S prodrugs and donors may serve as a crucial next step in drug development. With continued research efforts in this field, we believe H_2_S can play a vital role as a therapeutic for CVDs.
